# Cardiac Computed Tomography in Structural Heart Interventions: From Preprocedural Planning to Procedural Strategy

**DOI:** 10.31083/RCM46998

**Published:** 2025-12-24

**Authors:** Andreas Mitsis, Michaela Kyriakou, Artemis Fouseki, Kimon Myrianthopoulos, Maria Hadjicosti, Evi Christodoulou, Nikolaos PE Kadoglou, Christos Eftychiou

**Affiliations:** ^1^Cardiology Department, Nicosia General Hospital, State Health Services Organization, 2029 Nicosia, Cyprus; ^2^Radiology Department, Nicosia General Hospital, State Health Services Organization, 2029 Nicosia, Cyprus; ^3^Cardiology Department, Limassol General Hospital, State Health Services Organization, 4131 Limassol, Cyprus; ^4^Medical School, University of Cyprus, 2115 Nicosia, Cyprus

**Keywords:** computed tomography, structural intervention, transcatheter aortic valve implantation, mitral valve, tricuspid valve, left atrial appendage occlusion, septal defect, paravalvular leak

## Abstract

Cardiac computed tomography (CT) has become an essential imaging modality in structural cardiac interventions, providing high-resolution anatomical and functional assessments. Moreover, the role of cardiac CT spans pre-procedural planning, intra-procedural guidance, and post-procedural follow-up in interventions such as transcatheter aortic valve implantation (TAVI), mitral, tricuspid, and pulmonary valve interventions, left atrial appendage occlusion (LAAO), atrial septal defect (ASD), and paravalvular leak (PVL) closures. Furthermore, compared to traditional imaging techniques, cardiac CT offers superior spatial resolution, precise anatomical characterization, and improved procedural success rates by minimizing complications. Additionally, advances in artificial intelligence (AI)-driven CT analysis, perfusion imaging, and four-dimensional cardiac CT are expanding the associated applications. This review discusses the current role, benefits, limitations, and future perspectives of cardiac CT in guiding structural heart interventions.

## 1. Introduction

Over the last two decades structural cardiac interventions have transformed the management of valvular and congenital heart diseases, offering minimally invasive alternatives to traditional surgery [1]. As these interventions become more popular, the need for precise pre-procedural planning has become paramount. Advanced cardiac imaging plays a crucial role in optimizing procedural success, minimizing complications, and improving patient outcomes.

Computed tomography (CT) has emerged as a key imaging modality in structural interventions due to its high resolution, three-dimensional (3D) anatomical assessment, cost-effectiveness, broad and immediate availability, easy interpretation, as well as the ability to provide functional insights [2]. Compared to traditional imaging techniques such as transthoracic (TTE), transoesophageal echocardiography (TOE), cardiac magnetic resonance imaging (MRI) or fluoroscopy, CT offers a detailed visualization of cardiac anatomy, vascular access pathways, and prosthetic device positioning, making it an essential tool for interventional cardiologists.

This review explores the role of CT in various structural heart interventions, including transcatheter aortic valve implantation (TAVI), mitral and tricuspid valve transcatheter interventions, left atrial appendage occlusion (LAAO), atrial and ventricular septal defect (ASD/VSD) closures, pulmonary valve interventions and paravalvular leak (PVL) closures. Additionally, this manuscript compares CT with other imaging modalities, highlights its limitations, and provides a broad selection of tables and figures with multiple practical insights that clinicians can apply directly. Finally discusses emerging advancements, such as artificial intelligence (AI)-driven CT interpretation, CT-derived functional imaging, and low-dose protocols, providing clinicians and researchers with a comprehensive understanding of its benefits, challenges, and evolving applications in the field.

## 2. Fundamentals of CT for Structural Interventions

Contemporary multi-detector CT scanners offer excellent spatial and temporal resolution, providing precise visualization of cardiac chambers, valves, and vascular structures. A basic understanding of their technical principles and practical considerations is essential for accurate image acquisition and interpretation. For structural interventions, it is recommended to use at least a 64-slice CT scanner and a slice thickness of 0.6–0.75 mm [3], while newer models with more slices (e.g., 128, 256, or even 320) can offer faster scanning times and improved image resolution. Furthermore, electrocardiographic (ECG) gating is important for reducing motion artifacts, with protocol selection guided by patient rhythm, heart rate, and the need for functional information [4].

Adequate patient preparation is critical [5]. Heart rate control with β-blockers is generally tolerated and should be considered if heart rates are above 80 beats/min [6], while sublingual nitrates may be administered to improve vascular visualisation when synchronous assessment of coronaries might be needed [7]. Patients should be able to perform a brief breath-hold to avoid respiratory artifacts. The use of iodinated contrast material, with tailored bolus timing [8, 9], generally given at a rate of 4–6 mL/s, amounting to a total of 50–100 mL, ensures optimal opacification of the cardiac cavities and close vessels, but due to frequent renal comorbidities and the higher age of this group of patients, it should be applied with caution [10]. Achieving optimal contrast enhancement requires individualized adjustment of iodine dose and injection parameters, taking into consideration patient body size, iodine delivery rate, injection duration, and CT acquisition speed [11]. Of note, radiation exposure remains an important consideration; contemporary dose-saving techniques such as tube current modulation and iterative reconstruction are widely implemented to minimize risk while maintaining diagnostic quality [12, 13].

Post-processing imaging techniques play a central role in obtaining clinically relevant information. Multiplanar (MPR) and curved planar reformations (CPR) allow manual visualization in different planes (axial, sagittal, coronal, or oblique) from a single scan and require sufficient experience [14]. Conversely, 3D volume rendering using specific software products for image processing is used to characterize anatomy and to perform precise measurements [15]. Nonetheless, understanding of these fundamental principles ensures that cardiac CT can be applied effectively as the cornerstone of structural intervention planning.

### Limitations of Cardiovascular CT

Despite its strengths, cardiovascular CT is not without limitations. Radiation exposure remains a concern, particularly in younger patients and those requiring repeated imaging. Although modern scanners with iterative reconstruction techniques [16], dose modulation, and prospective ECG-gating have markedly reduced exposure, cumulative dose considerations persist in the context of surveillance imaging [17]. Contrast-related risks represent another limitation. Iodinated contrast administration can cause nephropathy in patients with pre-existing chronic kidney disease [18]. In addition, allergic or anaphylactoid reactions, though rare, can sometimes be expected and managed appropriately. Low-iodine protocols and iso-osmolar contrast agents may minimise some of these risks [19]. Arrhythmias and high heart rates can compromise image quality, even with ECG-gating. Patients with atrial fibrillation or frequent ectopic beats often present with motion artifacts and nondiagnostic images [20]. While advanced reconstruction algorithms and high temporal resolution scanners provide partial solutions, imaging in these groups remains technically challenging. Extensive calcification is a further limitation. Dense calcific burden may generate blooming artifacts that obscure anatomical borders, leading to inaccurate annular or vascular measurements [21]. This is particularly problematic in elderly patients with advanced valvular and vascular disease. Obesity and body habitus can significantly reduce image quality due to photon attenuation, requiring higher radiation doses or resulting in increased noise. Similarly, patients unable to perform adequate breath-holds may produce motion artifacts that limit image interpretation. Finally, access and availability are uneven across institutions. Cardiovascular CT requires advanced equipment, experienced operators, and specialized post-processing software. Variability in acquisition protocols and measurement techniques can introduce inter-observer differences, underscoring the importance of standardization and training [22]. Taken together, these limitations highlight the need for careful patient selection, optimization of imaging protocols, and close integration with complementary modalities such as echocardiography and MRI (Table 1).

**Table 1.** **Comparative strengths and limitations of CT, TOE, and MRI in structural heart interventions**.

Modality	Strengths	Limitations	Clinical role
CT	• Excellent 3D resolution	• Radiation exposure	• TAVI: annulus, root, coronaries
	• Reproducible measurements (annulus, leaflets, landing zones)	• Iodinated contrast risks	• MV/TV: annular sizing, leaflet tethering, landing zones, spatial relation to coronaries and conduction system
	• Superior for calcium burden, coronary proximity, peripheral access	• Motion artifacts with AF or high HR	• LAAO: ostium/landing zone
	• Comprehensive preprocedural planning for MV/TV interventions	• Blooming from heavy calcification	• ASD/VSD: defect morphology
		• Requires specialized software/expertise	• PVL: leak mapping
			• Pulmonary valve: RVOT and coronaries
TOE	• Real-time intraprocedural guidance	• Semi-invasive (anaesthesia/sedation)	• MV/TV: procedural guidance for edge-to-edge repair
	• Gold standard for LAA thrombus detection	• Operator dependent	• TAVI: guidance when echo-fusion used
	• High-resolution for leaflet motion, regurgitation jets, interatrial septum	• Limited 3D data	• LAAO: thrombus exclusion and intraprocedural guidance
	• Essential for MV and TV procedural guidance	• May underestimate annulus/device landing zones	• ASD/VSD: device deployment guidance
			• PVL: intra-procedural visualization
MRI	• Radiation-free	• Limited availability	• MV/TV: regurgitant volume quantification, RV function
	• Gold standard for ventricular volumes and function	• Long acquisition	• Congenital/pulmonary valve: RV volumes, regurgitant flow
	• Quantifies regurgitant fraction, Qp:Qs	• Contraindications: implantable devices, claustrophobia	• ASD/VSD: shunt quantification (Qp:Qs)
	• Tissue characterization (fibrosis, oedema)	• Suboptimal for calcium, prosthetic valves	• Pre-TAVI: limited, but useful if CT contraindicated
	• Useful for right-sided lesions (TV, pulmonary)		

AF, atrial fibrillation; ASD, atrial septal defect; CT, computed tomography; HR, heart rate; ICD, implantable cardioverter-defibrillator; LAAO, left atrial appendage occlusion; MRI, magnetic resonance imaging; MV, mitral valve; PVL, paravalvular leak; Qp:Qs, pulmonary-to-systemic flow ratio; RV, right ventricle; RVOT, right ventricular outflow tract; TAVI, transcatheter aortic valve implantation; TOE, transoesophageal echocardiography; TV, tricuspid valve; VSD, ventricular septal defect; 3D, three-dimensional.

## 3. CT in Specific Structural Interventions

### 3.1 Transcatheter Aortic Valve Implantation (TAVI)

Aortic stenosis is the most prevalent valvular disease in Western nations, primarily driven by age-related degenerative processes [23]. The use of TAVI has revolutionized treatment, initially reserved for high-risk surgical candidates but now extended to intermediate- and low-risk populations [24]. Optimal outcomes depend on meticulous preprocedural planning, with multi-slice CT established as the gold standard for the necessary anatomical assessment. While TTE and TOE remain important, CT provides a comprehensive 3D detail essential for transcatheter heart valve (THV) selection and procedural strategy [25]. Originally used for vascular access planning, CT has evolved into a multidimensional tool, providing a comprehensive assessment of the aortic root, valve morphology, and peripheral vasculature—key parameters that inform valve sizing and access planning (Table 2) [26].

**Table 2.** **CT-based predictors of TAVI complications**.

CT finding	Practical definition/measurement	Associated risk
Heavy annular or LVOT calcification	>20% annular circumference calcified or large nodules (>4–5 mm)	PVL, annular rupture, device under-expansion
Extremely large annulus	Area >680–700 mm^2^ or perimeter >32 mm	PLV, device migration or embolization
Extremely small annulus	Area <400 mm^2^ or perimeter <23 mm	PPM, subclinical valve thrombosis
Bicuspid valve with calcified raphe	Fused raphe with >3–4 mm thickness or severe, noncompliant calcification	Under-expansion, PVL, device migration
Narrow sinus of Valsalva	<30 mm (ideally >30–34 mm for safety)	Coronary obstruction, especially if cusp length > coronary height)
Sinotubular junction	Should exceed device size by ≥2 mm	Influences valve expansion and sealing
Low coronary ostial height	<12 mm (high risk), 12–14 mm (borderline)	Coronary obstruction, acute ischaemia
Iliofemoral artery minimal luminal diameter	≥5.5 mm for low-profile (14–16F) systems	Vascular injury, dissection, perforation
Severe iliofemoral tortuosity	>2 severe bends (>90°) or kinking with calcification	Access failure, vascular complications
Circumferential iliofemoral calcification	≥270° arc of calcification on axial images	Vessel rupture, dissection
Extensive aortic arch atheroma	Mobile/ulcerated plaque >4 mm thickness	Embolic stroke
Horizontal aorta	Aortic angulation >60° relative to annulus	Delivery difficulty, longer procedural time

LVOT, left ventricular outflow tract; PPM, patient prosthesis mismatch.

#### 3.1.1 Peripheral Arteries

**Fig. 1.**
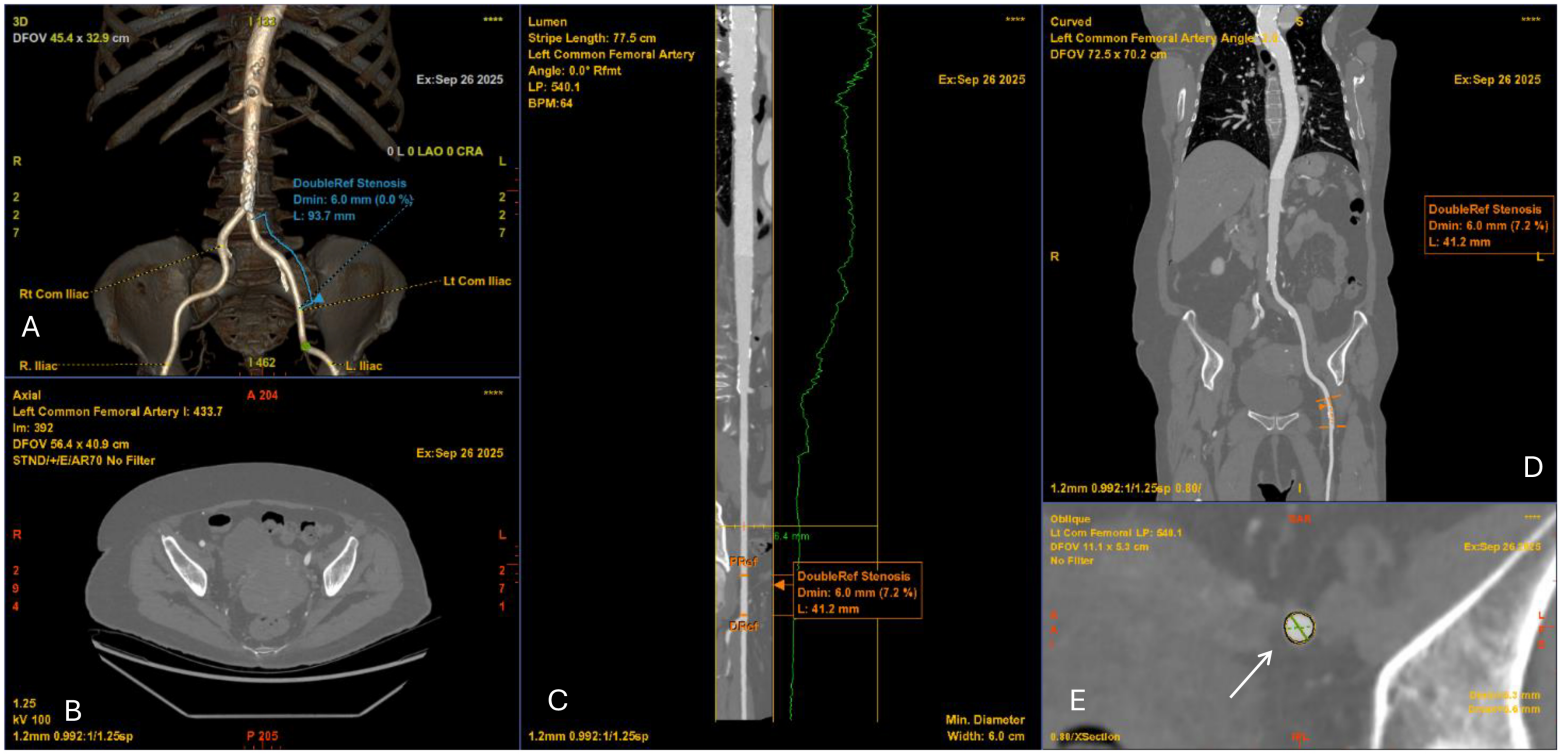
**Computed tomography angiography (CTA) of the iliofemoral vessels for TAVI access planning**. (A) 3D volume-rendered reconstruction of the iliofemoral arteries demonstrating vascular course and minimal luminal diameter of 6.0 mm. (B) Axial CTA slice at the pelvic level showing vessel lumen and surrounding structures. (C) Curved multiplanar reconstruction of the left common femoral artery with luminal profile (green line) demonstrating focal stenosis (minimal diameter 6.0 mm). (D) Coronal CTA reconstruction confirming the level of stenosis (6.0 mm). (E) Cross-sectional image at the site of stenosis (white arrow) with measurement of the minimal luminal area, relevant for determining vascular access feasibility.

Transfemoral access is preferred for TAVI whenever possible, making careful CT evaluation of the iliofemoral arteries essential [27]. CT provides accurate quantification of luminal diameter, calcific burden, tortuosity, and the presence of stenotic or aneurysmal disease (Fig. 1). CPR and centreline-based analyses should always be used to account for tortuosity [28]. The minimal luminal diameter must be measured precisely. However, vessel suitability is not defined by diameter alone. Other factors such as circumferential calcification and tortuosity, can significantly increase the risk of dissection or perforation, even if the lumen size is adequate [29]. Tortuosity grading further informs procedural planning, as severe angulation can impede sheath advancement [30, 31]. Despite lower-profile delivery systems and improved closure devices [32], thorough vascular evaluation remains necessary to reduce vascular-related complications such as dissection, perforation, or occlusion [33]. When anatomy is unfavourable including small calibre, concentric calcification, or extreme tortuosity, alternative access routes should be considered [34].

#### 3.1.2 Aorta

**Fig. 2.**
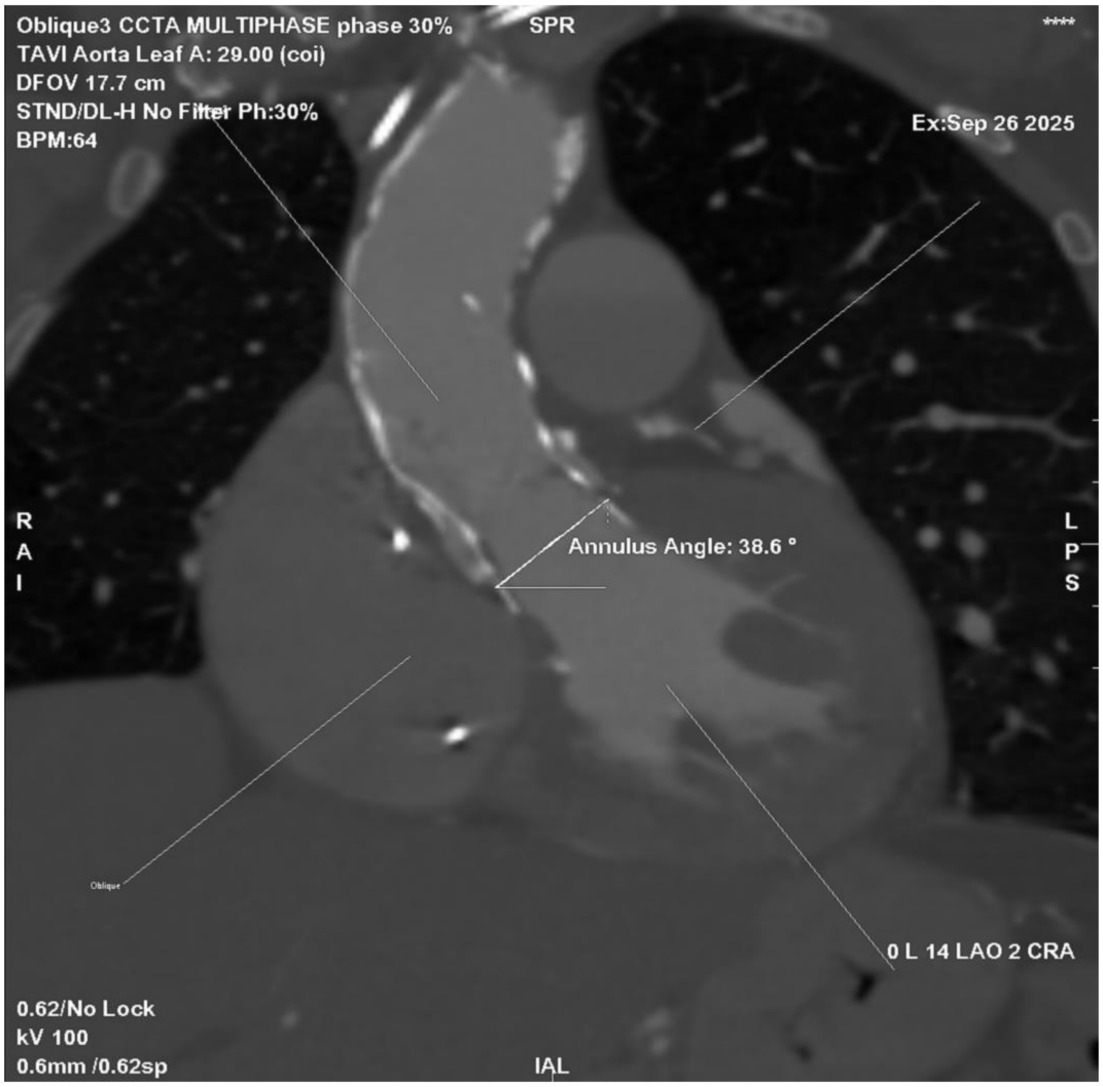
**Cardiac CT angiography (CCTA) showing aortic root angulation in the planning of TAVI**. Multiplanar reconstruction at 30% of the R–R interval demonstrates an annular angulation of 38.6°, measured between the aortic annulus plane and the horizontal reference line. Accurate assessment of aortic angulation is critical for selecting the optimal fluoroscopic projection and minimizing parallax during valve deployment.

Evaluation of the thoracic and abdominal aorta is equally important. Ulcerated, eccentric, or mobile atheroma in the ascending aorta or arch markedly increases the risk of embolic stroke during catheter manipulation. Detailed visualization of plaque morphology can trigger embolic protection strategies [35, 36]. Furthermore, the management of patients with aortic disease, including abdominal aortic aneurysms (AAA) or previous endovascular aortic repair (EVAR) remains challenging [37]. Other aortic procedures, such as ascending aortic replacement or arch reconstruction, may also influence the feasibility and risk of TAVI. A precise visualisation of the aorta pathology with CT scan, excluding residual dissections, penetrating aortic ulcers or incomplete thrombosis of the false lumen, is crucial to determine the optimal approach for TAVI and to minimize potential complications [38]. Another important characteristic that requires attention is the aortic angulation, which is defined as the angle between the horizontal plane and the aortic annulus plane in a coronal projection [39, 40]. The degree of this angulation can affect the precise positioning of the THV during TAVI making the procedure more challenging (Fig. 2), particularly in an extremely angulated or horizontal aorta (HA) [34]. HAs are often seen in elderly patients, complicating THV passage and should be recognized during planning, particularly for balloon-expandable valves (BEVs) [41]. Finally, the presence of suprarenal atheroma requires consideration because it has been linked to acute kidney injury following TAVI, likely due to increased embolic and contrast load [42, 43]. Thus, systematic characterization of aortic pathology is required to balance access strategy and protection measures.

#### 3.1.3 Sinotubular Junction (STJ)

**Fig. 3.**
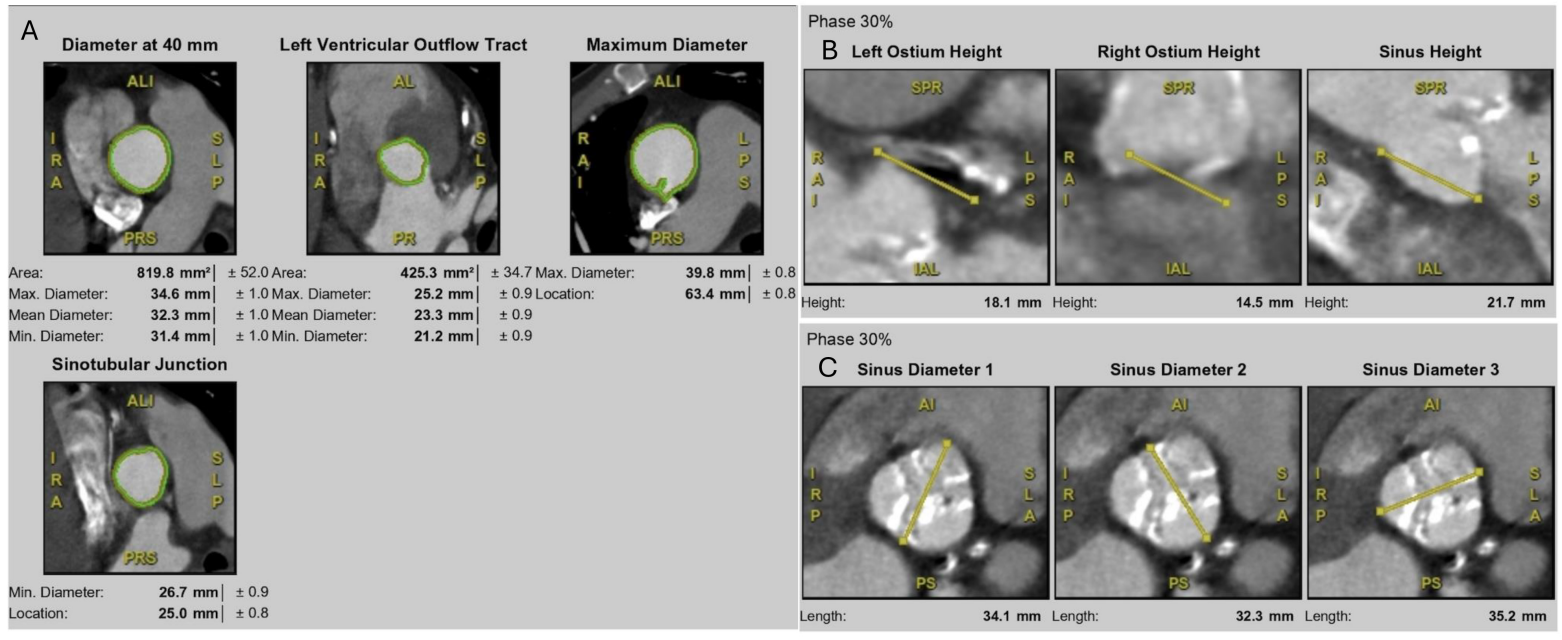
**Multidetector CT measurements for TAVI planning**. (A) Aortic root assessment at multiple levels, including annulus (diameter at 40 mm), LVOT, sinotubular junction (STJ), and maximum aortic root diameter. (B) Coronary ostium height measurements at 30% cardiac phase, including left and right coronary ostia and sinus height. (C) Sinus of Valsalva dimensions at 30% cardiac phase, showing three sinus diameters relative to the annular plane.

The STJ, forming the outflow boundary of the aortic root, plays a critical role in THV deployment. A narrow or calcified STJ relative to the sinuses may lead to suboptimal hemodynamic performance, while a large, tapered STJ may compromise anchoring and increase long-term the risk of leaflet thrombosis [44, 45]. BEVs are particularly sensitive to STJ constraints, whereas self-expanding valves (SEVs) present better accommodation variability. In BEVs, interaction between the deployment balloon or stent frame and calcification at the STJ can increase the risk of balloon rupture or aortic root injury. Accordingly, assessment of the STJ area and height is essential in all candidates (Fig. 3). A high and spacious STJ relative to the intended valve size is generally favourable for TAVI, whereas a low, narrow, and calcified STJ poses significant technical challenges. In such cases, the use of a shorter-frame THV that can be positioned below the level of calcification may be preferable [45]. Careful CT measurement of the STJ diameter and its relationship to the sinus of Valsalva is therefore essential to guide valve selection.

#### 3.1.4 Sinus of Valsalva and Coronary Ostia 

Coronary obstruction, though rare, is a catastrophic complication [46]. CT-derived measurements of sinus of Valsalva width and coronary ostial height can identify patients at increased risk, particularly when cusp length exceeds coronary height [47]. In such patients, preventive measures such as coronary protection, Bioprosthetic or Native Aortic Scallop Intentional Laceration to Prevent Iatrogenic Coronary Artery Obstruction (BASILICA) leaflet laceration, or alternative valve platforms should be considered [48, 49]. Importantly, both coronary arteries must be assessed individually, as asymmetric sinus anatomy may disproportionately endanger one ostium.

#### 3.1.5 Aortic Root and Annulus

**Fig. 4.**
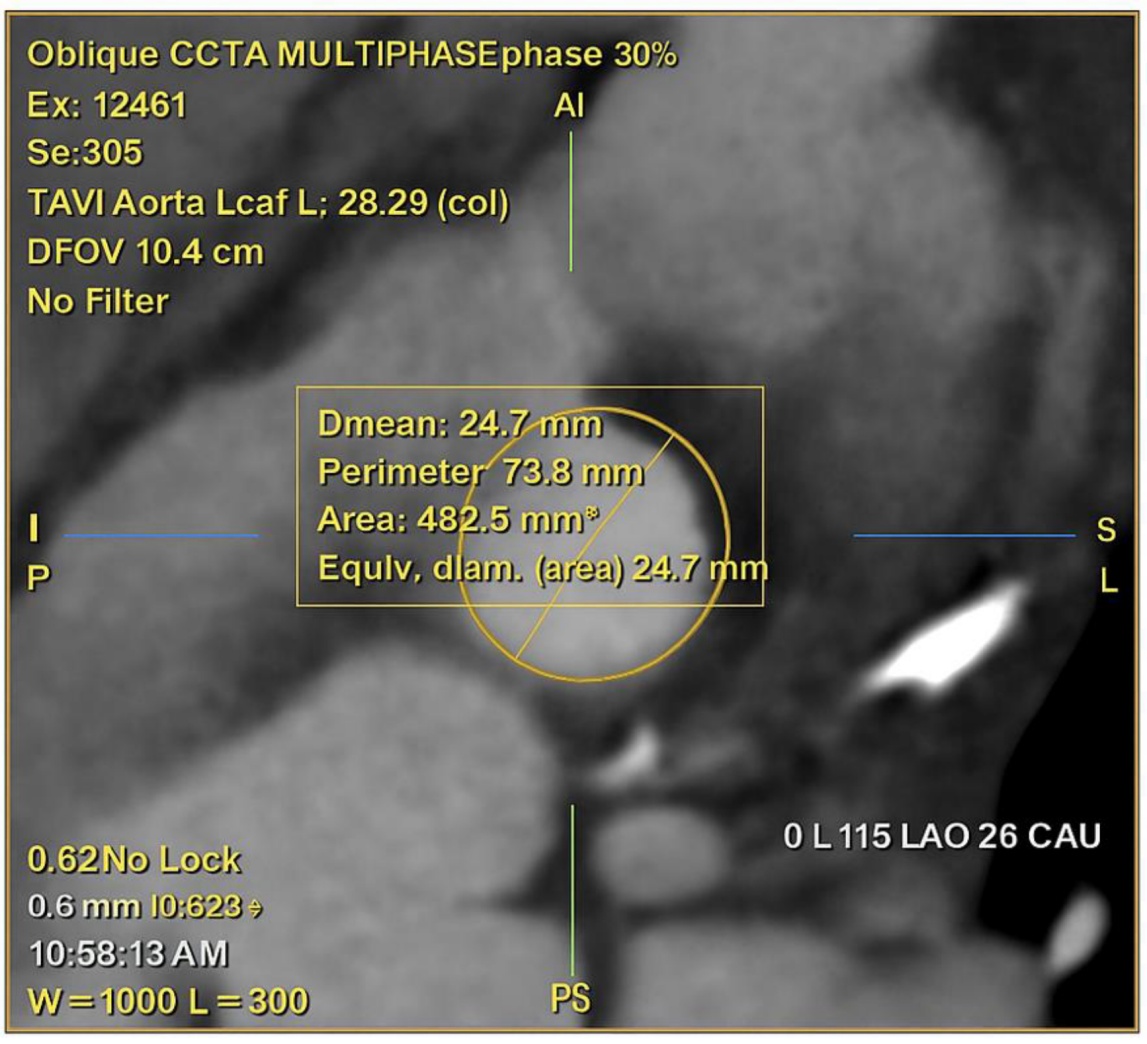
**CCTA with multiplanar reconstruction at 30% of the R–R interval, showing oblique annular plane measurements for TAVI**. The mean annular diameter is 24.7 mm, corresponding to an area of 482.5 mm^2^ and a perimeter of 73.8 mm.

Accurate annular sizing is the cornerstone of THV selection (Fig. 4). The aortic annulus, anatomically defined by a virtual ring connecting the basal hinge points of the cusps [50]. The annulus should be measured in systole (20–40% of the R–R interval), when dimensions are largest and most reproducible. CT defines the “virtual annulus” allowing calculation of area, perimeter, and diameters [51]. Area- and perimeter-derived sizing is more reliable than single diameters, particularly in elliptical annuli. Device selection generally involves 5–15% oversizing to minimize PVL while avoiding annular rupture. Therefore, incorrect sizing carries severe consequences as under-sizing contributes to PVL and device migration, whereas oversizing risks annular rupture, especially in heavily calcified rings [52].

#### 3.1.6 Device Implantation Zone

The implantation zone includes the annulus, cusps, and left ventricular outflow tract (LVOT). Calcification within this zone must be described not only by volume but also by distribution. The presence of annular or LVOT calcification is known to increase the risk of adverse outcomes with TAVI [53]. Concentric calcium may provide anchoring, but bulky nodules in the LVOT substantially increase the risk of rupture if a balloon-expandable device is chosen. Conversely, asymmetric commissural calcium may predispose to paravalvular leak [54]. Therefore, CT assessment is crucial to guide the THV selection. SEVs may be favoured in heavily calcified annuli with the presence of multiple nodules of calcification of a single focus extending >10.0 mm in length or covering >20% of the perimeter of the annulus and in cases of heavily calcified LVOTs due to their lower radial force and lower risk of rupture. Conversely, BEVs can achieve better sealing in asymmetric calcium patterns [55].

#### 3.1.7 Valve Morphology and Raphe Calcification

In bicuspid aortic valves, CT is mandatory for defining valve morphology, commissural orientation, and raphe calcification. Severe raphe calcification increases the risk of under-expansion and incomplete sealing [56]. Furthermore, commissural alignment between the prosthesis and native anatomy has become a focus of contemporary practice, as it preserves coronary access for potential future interventions [57]. CT provides reproducible measurements that predict technical difficulty, for example, cases with fused raphe often require balloon pre-dilatation to fracture calcified bridges before valve expansion [58].

#### 3.1.8 Valve in Valve TAVI

**Fig. 5.**
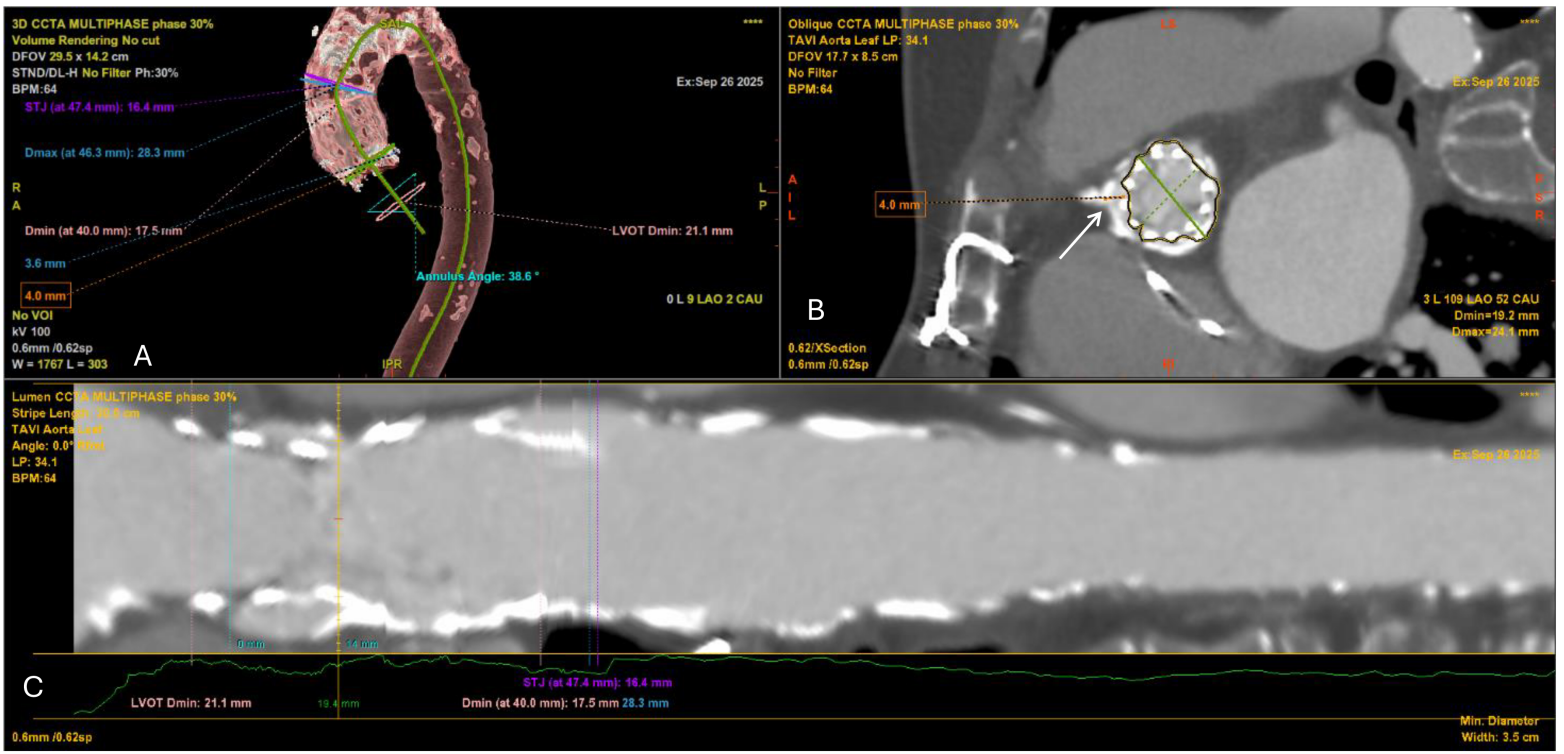
**Multidetector CT angiography for preprocedural planning of valve-in-valve TAVI**. (A) 3D volume-rendered reconstruction of the thoracic aorta and aortic root showing the previously implanted surgical aortic bioprosthesis (SAVR) *in situ*, the heavily calcified ascending aorta, and measurements of the aortic annulus, LVOT and STJ. The annular angulation relative to the long axis of the aorta is also depicted. (B) Oblique cross-sectional view at the level of the surgical bioprosthesis demonstrating the internal stent frame and leaflets. The white arrow indicates the virtual transcatheter valve-to-coronary (VTC) distance to the right coronary ostium (measured at 4.0 mm), which is critical for assessing the risk of coronary obstruction. (C) Curved multiplanar reconstruction (centerline analysis) of the aortic root and ascending aorta, illustrating luminal diameters along the LVOT, annulus, and STJ. Minimal and maximal diameters are annotated, confirming the constrained annular geometry imposed by the prior surgical valve.

Valve in Valve (ViV) TAVI is considered as a valid therapeutic option in patients with degenerated bioprosthetic surgical heart valves (SHVs) [59, 60], or previous TAVI, especially in patients with high operative risk [61]. Estimating the risk of coronary artery occlusion, as well as knowing the surgical heart valve type and size is crucial in ViV cases [62]. Pre-procedural CT is the gold standard. The decisive parameter for a ViV procedure is the distance between the ostia of the coronaries and the expected final THV position. Simulating a virtual ring, which represents the expanded THV, aligned geometrically with the surgical valve is performed using pre-interventional CT imaging analysis. The distance between this virtual ring and the ostia of the coronary arteries, i.e., the VTC (Virtual THV to coronary distance) as well as Valve to STJ (VTSTJ) distances are essential parameters that need to be calculated to justify the feasibility of the procedure (Fig. 5). Especially for the risk of coronary ostia occlusion 3 to 6mm represents intermediate risk and <3–4 mm represents high risk cases [63]. If the VTC is ≤4 mm or the culprit leaflet calcium volume is >600 mm^3^, either surgery or BASILICA should be considered [64]. Snorkel stenting may be considered in palliative cases when the risk of stent thrombosis and lack of future coronary access may be acceptable [65].

The SHV type (stented, stentless, sutureless) and size (in cases of unclear surgical history) can be distinguished using CT analysis, as well as high-risk features for coronary occlusion during the procedure, such as bulky calcifications, pannus, failed prostheses, and leaflet presence [66]. Finally, providing a detailed anatomic view utilizing MPR is extremely helpful in pre-intervention planning. Of note, in patients with renal impairment, radiopaque parts of the surgical valve, as well as ostia of the coronaries can be identified without contrast [67].

#### 3.1.9 Role of Cardiac CT in Post-TAVI Surveillance

**Fig. 6.**
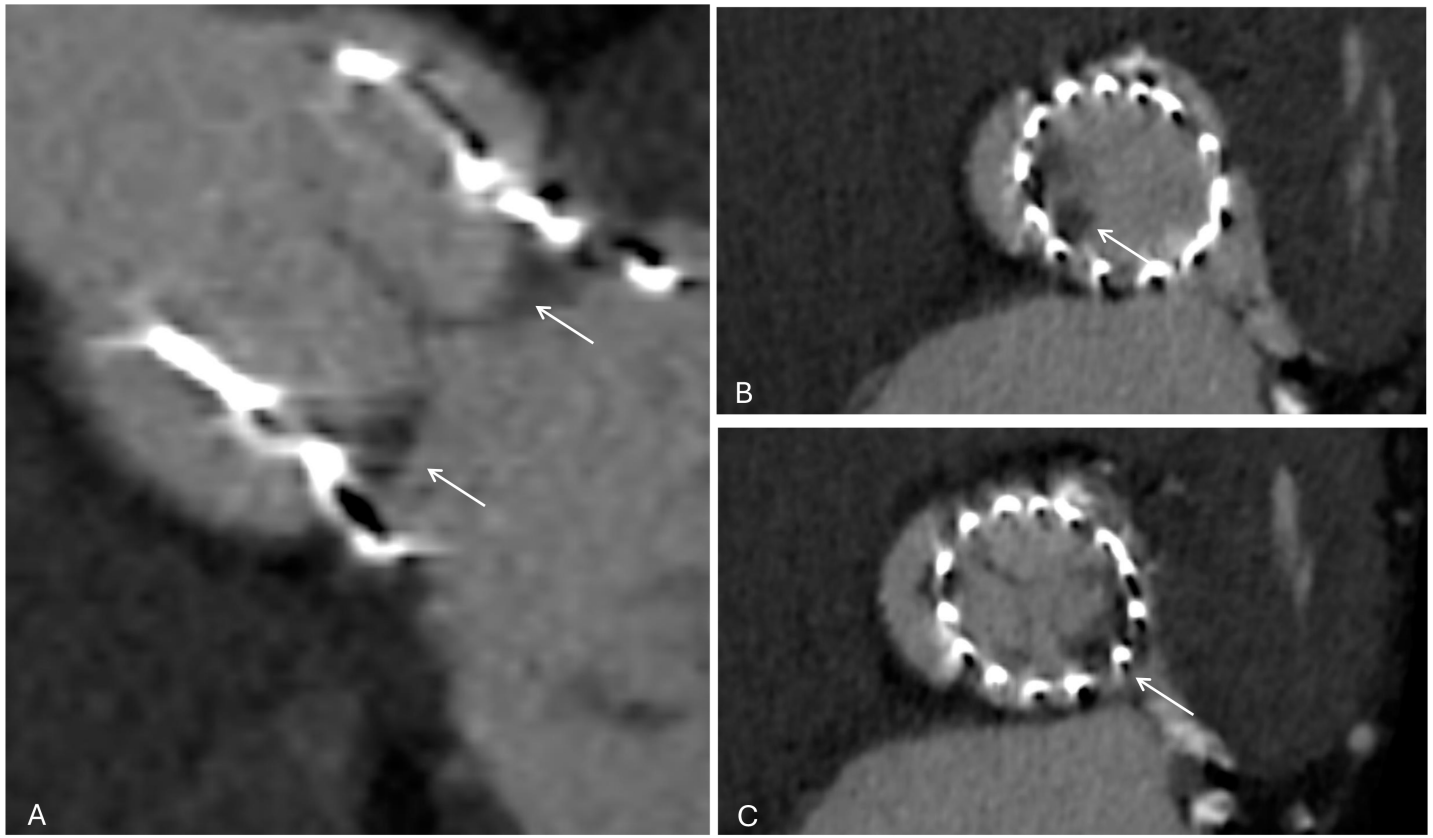
**Cardiac CT demonstrating hypo-attenuated leaflet thickening (HALT) after TAVI**. (A) Short-axis view of the transcatheter valve (white arrows). (B) At the mid-leaflet level, HALT is visible in the non-coronary cusp (white arrow). (C) At a slightly different reconstruction plane, HALT is instead visualized in the left coronary cusp (white arrows). These findings highlight how leaflet thrombosis can involve different cusps and may only be appreciable at specific imaging planes.

Cardiac CT has become the gold standard imaging modality for post-TAVI evaluation. It allows precise assessment of prosthesis expansion, leaflet motion, paravalvular leaks, and coronary ostia patency. Importantly, CT can detect hypo-attenuated leaflet thickening (HALT), a manifestation of subclinical leaflet thrombosis that may not be apparent on echocardiography [68]. Careful multiplanar review is essential, as HALT may be missed if only a single imaging plane is evaluated (Fig. 6). Beyond HALT, CT also quantifies calcium burden, evaluates stent frame position, and helps in planning potential re-intervention. Thus, CT provides comprehensive structural and functional insights critical for long-term surveillance of TAVI patients.

### 3.2 Transcatheter Mitral Valve Interventions

CT has become an essential tool in the planning of transcatheter mitral valve interventions and especially in cases of transcatheter mitral valve replacement (TMVR) [69]. CT enables accurate quantification of mitral annular dimensions, perimeter, and non-planarity, as well as evaluation of leaflet tethering, sub-valvular apparatus, and chamber volumes. This information is important for device sizing, patient selection, and procedural strategy. In addition, CT describes anatomic contributors to LVOT narrowing, such as the anterior mitral leaflet or basal septum, and can help predict the neo-LVOT area and assess the risk of LVOT obstruction [70]. Beyond valve-specific assessment, CT can be used to simulate procedural fluoroscopic angles and evaluate the atrial septum for transseptal access (Table 3).

**Table 3.** **CT for mitral and tricuspid interventions — what to measure, how, and why**.

Measurement (CT)	How to measure (acquisition/plane)	Practical thresholds & typical values	Why it matters (planning/strategy)
Mitral annulus (TMVR)	3D MPR; D-shaped annulus in mid-to-late diastole	Area, perimeter; D-shape excludes anterior horn	Correct sizing → prevents embolization (undersizing) or rupture (oversizing)
Mitral annular calcification (MAC)	Axial and short-axis MPR; calcium scoring	Severe MAC = score ≥7; nodular vs diffuse	Determines feasibility of ViMAC; circumferential calcium anchors device, nodular causes PVL/embolization
Landing zone (TMVR/ViMAC)	3D annular reconstruction; use circumflex artery as landmark	80% ventricular/20% atrial offset typical	Defines anchoring site; calcification distribution and ridges influence stability
Neo-LVOT prediction	Virtual valve implantation at end-systole (≈40% R–R)	High risk if neo-LVOT <200 mm^2^	Identifies obstruction risk; guides device choice, septal reduction strategies
Tricuspid annulus	Short-axis CT at end-diastole; align with 4- and 2-chamber views	Normal ~3.0–3.5 cm; dilated >4.0 cm	Prosthesis sizing; large annuli may exceed device range
Leaflet tethering (TR severity)	Measure tenting height/area from annular plane to coaptation	Increased tethering height (>8–10 mm) or area	Indicates RV remodeling; predicts residual TR after repair/replacement
Right coronary artery proximity	Distance annulus → RCA on axial/coronal MPR	<2 mm = unfavorable	Guides risk of coronary compression during annuloplasty/TTVR
Conduction system (septal leaflet/His bundle)	Distance from septal annulus to membranous septum	<2–3 mm = higher risk	Predicts AV block; influences device anchoring strategy
RV size & geometry	End-systolic RV length/diameter on axial & 4-chamber	Axial diameter ≥60 mm (men), ≥57 mm (women)	RV dilation = advanced disease; influences device feasibility and outcomes

MPR, Multiplanar; ViMAC, valve-in-MAC; RCA, right coronary artery.

In opposite, the use of CT in transcatheter-edge-to-edge-repair (TEER) is rare. 3D-TOE is considered the gold standard for pre-procedural planning and peri-procedural success. However, in challenging cases CT can help define mitral annular dimensions, leaflet length, and the spatial relationship to nearby structures, complementing echocardiographic assessment of leaflet grasping zones and regurgitant jet location [71].

#### 3.2.1 Mitral Annulus Assessment

Prior to TMVR, accurate CT-based sizing of the mitral valve apparatus is critical, as under-sizing may lead to paravalvular leak or embolization, whereas oversizing may cause rupture. Because annular dimensions vary dynamically, measurements should be obtained in both systole and diastole [72], but sizing is conventionally performed in mid-to-late diastole when the annulus is maximal [73]. Given the challenges of reconstructing the true 3D saddle-shaped geometry, a simplified two-dimensional D-shaped annulus is often used, providing a reproducible framework for device sizing [74]. Importantly, if the saddle-shaped annulus is used for sizing, its planar projection extends into the LVOT, potentially overestimating the landing zone and increasing the risk of obstruction [75]. Conversely, the D-shaped annulus, by excluding the anterior horn—which does not contribute to prosthetic anchoring—offers a more accurate representation of the true landing zone.

#### 3.2.2 Calcification Quantification

Severe mitral annular calcification (MAC) defines a high-risk patient group often excluded from early TMVR trials [76]. The valve-in-MAC (ViMAC) approach has since emerged as a potential option for these patients, though it remains technically challenging and carries higher complication rates. In planning ViMAC procedures, careful assessment of calcification extent, morphology and distribution is crucial, with particular attention to spurs—calcific protrusions that may hinder valve seating or cause obstruction—and gutters, which are gaps from irregular calcification that predispose to paravalvular leak [77]. MAC distribution can be categorized as circumferential or noncircumferential, with circumferential involvement offering the most favorable anchoring conditions for TMVR [77]. Annular calcium may be inelastic with elevated embolic risk, soft, with poor anchoring, or dense allowing anchoring but limiting expansion and risking injury. A CT-derived scoring system has been proposed to grade MAC severity and help predict THV embolization in ViMAC procedures [78]. The MAC score (0–10), incorporating calcium thickness, circumferential extent, and involvement of commissures and leaflets, defines severe MAC at ≥7. Patients with a score ≤6 exhibited a substantially higher risk of valve embolization or migration compared with those scoring ≥7 (60% vs. 9.7%; *p* < 0.001) [78].

MAC may present as either nodular or diffuse disease, with equally important implications for ViMAC interventions. Nodular MAC is characterized by focal, bulky deposits that protrude into the annular space; these calcific nodules can interfere with valve seating, create paravalvular leaks, and increase the risk of prosthesis embolization or migration due to the lack of a continuous anchoring surface [79]. In contrast, diffuse MAC is defined by broad, circumferential involvement of the annulus, which generally provides a more uniform and stable landing zone for THVs and is associated with lower embolization rates [78]. However, diffuse circumferential calcification can also hinder full valve expansion, elevate transmitral gradients, or exacerbate the risk of left ventricular outflow tract obstruction. In addition, solid MAC provides more anchoring than caseous MAC, which has a core of liquefactive necrosis [80]. Thus, differentiating between nodular and diffuse patterns based on a proper CT assessment, is essential for pre-procedural planning and patient selection in ViMAC [81].

#### 3.2.3 Landing Zone 

The landing zone—defined as the area where the mitral device is deployed and includes the mitral annulus, ventricular and nearby supporting structures [82]. Importantly, the exact definition of the landing zone varies according to the TMVR device used [83]. For TMVR in the native mitral annulus, device measurements are obtained at the atrioventricular junction—where the left atrium meets the left ventricle—using the course of the circumflex artery along the posterior atrioventricular groove as an anatomic landmark [73]. Typically, a landing zone with 80% ventricular offset and 20% atrial offset is used for TMVR devices. In ViMAC procedures, anchoring is determined by the extent and distribution of calcification. The device landing zone is typically more ventricular, located at the waist of the calcification, where maximal radial constraint provides secure prosthesis fixation [84]. Several anatomic features, except the extent and distribution of MAC, contribute to defining an appropriate landing zone, including the presence of a fibromuscular ridge beneath the posterior leaflet that may offer an additional surface for device support and mitral annular disjunction (MAD), a separation between the posterior annulus and left ventricular myocardium that may compromise anchoring stability [85].

#### 3.2.4 Prediction of the Neo-LVOT

The LVOT is located between the basal interventricular septum anteriorly and the aortomitral continuity posteriorly. LVOT obstruction is the most serious complication of TMVR and is defined as a postprocedural gradient increase of ≥10 mmHg [86]. It occurs more frequently in valve-in-MAC cases (≈11.2%) [87] than in native valve replacement (0–1%) [88]. Following implantation, the TMVR device displaces the anterior mitral leaflet (AML) toward the septum, creating a “neo-LVOT” confined by the displaced AML, the prosthetic stent, frame and the basal to mid anteroseptal LV wall [89]. The neo-LVOT represents the minimal cross-sectional area of the left ventricular outflow tract expected to remain unobstructed after deployment of a TMVR prosthesis within the mitral annulus [90].

End-systole is optimal for neo-LVOT assessment, typically measured at ~40% of the cardiac cycle on gated cardiac CT to capture the narrowest dimension [90]. The neo-LVOT is derived from CT-based virtual valve implantation, where a 3D prosthesis is aligned to the mitral annulus. At end-systole, a three-chamber view is used to generate a short-axis reformation of the neo-LVOT, from which the minimal cross-sectional area is measured. Virtual THV sizing is based on the projected mitral landing zone. In valve-in-ring and valve-in-MAC, the device is positioned about 80% ventricular and 20% atrial depth [91]. For ViV TMVR, the virtual valve is modelled as a cylinder matching the proposed device dimensions, first flush with the surgical valve (0%) and then with a 20% ventricular extension [90]. In their landmark study validating neo-LVOT prediction after TMVR, Wang *et al*. [91] demonstrated a strong correlation between preprocedural CT-based modelling and postprocedural measurements (R^2^ = 0.82; *p* < 0.0001), thereby establishing cardiac CT as the reference standard for predicting LVOT obstruction risk.

### 3.3 Transcatheter Tricuspid Valve Interventions

Imaging the tricuspid valve (TV) with CT presents several unique challenges compared with the mitral valve. The valve’s anterior location can reduce contrast resolution and increase susceptibility to motion artifacts [92]. Its leaflets are thin, mobile, and often difficult to visualize, which limits the ability to assess morphology with the same accuracy achievable in the mitral position. The tricuspid annulus is large, saddle-shaped, and highly dynamic, making reproducible measurements across the cardiac cycle more complex [92]. In addition, the valve’s proximity to the right coronary artery and atrioventricular conduction tissue requires careful assessment to anticipate potential procedural complications [93]. A slice thickness less than 0.75 mm is usually preferred for better analysis, while the ideal dose modulation should be switched off to allow for data acquisition with peak tube current throughout the entire cardiac cycle. Notably, the frequent presence of cardiac implantable electronic device leads traversing the tricuspid valve may obscure leaflet anatomy, introduce artifacts, and hinder accurate reconstruction [94].

Orthotopic transcatheter tricuspid valve replacement (TTVR) systems such as EVOQUE bioprosthesis (Edwards Lifesciences) require CT-based quantification of annular area, perimeter, and diameters for prosthesis sizing and feasibility assessment. The EVOQUE bioprosthesis is available in 44-, 48-, 52-, and 58-mm sizes, with cardiac CT serving as the primary modality for screening and procedural planning [95]. Measurements are performed in end-diastole, with feasibility thresholds suggesting a perimeter-derived annular diameter (PDD) of 36.5–53.8 mm as optimal, while diameters >62 mm, PDD >57.5 mm or projected perimeters >180.5 mm are predictive of screening failure [96]. Notably, registry data report a high rate of TTVR screening exclusions, most often due to CT-defined anatomic factors such as excessive annular size, the presence of intracardiac leads, or small right-heart chambers [97].

#### 3.3.1 Leaflet Anatomy

The TV apparatus consists of the leaflets, annulus, chorda tendineae, and papillary muscles, with considerable anatomic variation. The normal configuration (Type I) has three leaflets—anterior, posterior, and septal—seen in ~54% of individuals. Variants include Type II (~5%), with fused anterior and posterior leaflets; Type III (~39%), with four leaflets, usually two posterior; and the rare Type IV (~2%), with five leaflets. Cardiac CT enables precise delineation of leaflet morphology, showing the anterior leaflet as the largest and most mobile, the septal as the shortest and least mobile, attached to the interventricular septum and the posterior as having the smallest circumferential extent. In normal function, the TV leaflets coaptation at or below the annulus during systole, with a coaptation length of 5–10 mm [98].

#### 3.3.2 Leaflet Thickness, Tethering, and Mobility

CT allows quantitative assessment of tricuspid leaflet geometry beyond simple annular dimensions. Parameters such as leaflet tethering height (the distance from the annular plane to the leaflet coaptation point) and tethering area (the area enclosed between the leaflets and the annular plane) provide valuable insight into the mechanism and severity of functional TR [99]. Increased tethering height and tenting area reflect right ventricular remodelling and papillary muscle displacement, both of which restrict leaflet motion and impair coaptation. Leaflet thickness can be accurately assessed by CT, with thickened or calcified leaflets often seen in rheumatic heart disease, carcinoid syndrome, or prior endocarditis [100].

#### 3.3.3 Anatomic Variants (Clefts, Fusion)

CT is essential in the pre-procedural assessment of TV anatomy, particularly for detecting anatomic variants such as leaflet clefts and commissural fusion. Leaflet clefts, which appear as slit-like separations within a leaflet, may mimic additional commissures and can reduce effective coaptation length, generate eccentric regurgitant jets, and complicate leaflet grasping during TEER. CCT, with its 3D resolution, facilitates differentiation of true commissures from clefts or indentations, a distinction that is often challenging with echocardiography. This capability is particularly relevant for guiding device positioning and assessing leaflet interaction during TTVR [101]. Commissural or leaflet fusion, more commonly associated with rheumatic involvement, endocarditis, or prior surgical intervention, is characterized on CT by loss of normal separation between adjacent leaflets with thickened or fibrotic tissue, leading to restricted motion and impaired orifice geometry. Accurate identification of these variants with multiplanar and 3D CT reconstructions is critical for device selection, procedural planning, and predicting the likelihood of residual regurgitation following TTVR.

#### 3.3.4 Relationships With Nearby Structures 

CT also plays a key role in assessing nearby structures that may be at risk during TTVR. The right coronary artery courses near the anterior and posterior annulus, making it susceptible to compression or injury during annuloplasty or device anchoring; a distance <2 mm from the annulus, most often near the posterior leaflet, is considered unfavourable [102]. Furthermore, the atrioventricular node and His bundle lie next to the septal leaflet on the membranous septum, predisposing to conduction disturbances and atrioventricular block during TTVR [103]. Also, the anteroseptal commissure lies nearby to the noncoronary sinus of Valsalva, device anchoring in this region carries a potential risk of aortic root perforation [104].

#### 3.3.5 Annular Dimensions

The tricuspid annulus dimensions are assessed on reconstructed short-axis images acquired at end-diastole, when the annulus reaches its maximal size following atrial contraction with the imaging plane manually aligned to the annular level on both four- and two-chamber views [103]. In the four-chamber view on two-dimensional (2D) echocardiography, normal tricuspid annular measurements are approximately 3.1 ± 0.4 cm in diameter, 11.9 ± 0.9 cm in circumference, and 11.3 ± 1.8 cm^2^ in area. In short-axis CT, the normal annular diameter is typically reported between 3.0 and 3.5 cm. In functional TR, annular dilatation is defined as a diameter >4.0 cm, with predominant enlargement of the anteroposterior and lateral dimensions and loss of saddle-shaped geometry [105].

#### 3.3.6 Right Ventricular Size and Geometry

Assessment of RV morphology is essential in planning TTVR. The RV has a complex shape, appearing triangular in the longitudinal plane and crescentic in cross-section as it wraps around the LV [106]. On CCT, RV enlargement is suggested by a transverse axial diameter of ≥60 mm in men and ≥57 mm in women [107]. RV length can also be measured at end-systole from the tricuspid annulus to the apex, with attention to anatomic structures along this course, such as papillary muscles and the moderator band [102].

### 3.4 Left Atrial Appendage Occlusion (LAAO)

Left atrial appendage occlusion (LAAO) is gaining momentum in the prevention of thromboembolic events in patients with atrial fibrillation (AF) who are unsuitable for long-term anticoagulation [108]. Procedural success and safety depend on accurate imaging assessment of the LAA, both before and after device implantation. Traditionally, TOE has been the major method of pre-procedural scanning; however, more recently cardiac CT has been proven more accurate and useful [109], as it provides superior 3D anatomic details and is associated with shorter total procedure time and a lower rate of device size change [110]. Nowadays, in many centres, CT is considered the primary, imaging modality for LAAO planning (Tables 4,5).

**Table 4.** **CT measurements for LAAO planning**.

Measurement (CT)	How to measure (acquisition/plane)	Practical thresholds & typical values	Why it matters (planning/strategy)
Thrombus vs slow flow	ECG-gated arterial + delayed phase (60–90 s); ROI in LAA cavity vs LA body	Persistent hypoattenuation with HU <~100 on delayed or large HU gap vs LA body → thrombus likely; resolution on delayed → slow flow	Thrombus → defer procedure/anticoagulated; slow flow → proceed with standard planning
Ostium diameter (max/min & mean)	True-ostium plane using LA–LAA junction; align by 3D MPR (use ridge/LSPV limbus/LCX as landmarks)	Common range 18–32 mm; report max, min, mean	Guides disc/cap sizing; larger ostia favor devices with broader sealing discs
Landing-zone diameter	Plane 10–12 mm distal to ostium along LAA centerline	Use mean diameter for sizing; plan device oversizing 10–30% (device-dependent)	Primary input for lobe/cap size (e.g., Watchman FLX, Amulet)
LAA depth	Perpendicular distance from ostium plane to the apex of the dominant lobe	Aim for ≥10–12 mm minimum; more if large device anticipated	Insufficient depth risks protrusion or instability; may favor disc-anchored designs
LAA morphology	3D review (MPR); note dominant lobe, secondary lobes, angle to LA	Chicken-wing, windsock, cactus, cauliflower	Complex/multilobed or shallow depth → prefer devices with robust disc coverage (e.g., Amulet/LAmbre)
Landing-zone ellipticity	Ratio short/long axis at landing zone	<0.8 = marked ovality	Oval landing zones benefit from higher oversizing and devices that tolerate ellipticity
Angulation	Angle between LA body and LAA centerline	Sharp angles (>45–60°) increase delivery difficulty	Consider sheath selection, support wires; disc-anchored devices may seat more predictably
Proximity to adjacent structures	Measure to MV, LUPV, LCx, left superior PV ridge	Distance ≥3–5 mm desirable	Prevents device impingement on MV/PV; adjust depth and orientation
IAS & access	CT of IAS and femoral/iliac veins (if available)	Favorable femoral venous route: IAS thickness typically <3–4 mm	Predict transseptal angle; may influence sheath curve selection
Peripheral venous access	Same dataset if whole-chest CTA; otherwise dedicated	Vein caliber ≥6–7 mm for large sheaths (center-specific)	Confirms feasibility and side selection for venous access

ECG, electrocardiogram; HU, Hounsfield unit; IAS, interatrial septum; LA, left atrium; LAA, left atrial appendage; LAAO, left atrial appendage occlusion; LCX, left circumflex artery; LSPV, left superior pulmonary vein; MV, mitral valve; OAC, oral anticoagulation; PV, pulmonary vein; ROI, region of interest.

**Table 5.** **LAAO CT-driven device planning and follow-up**.

Decision Point	CT-Guided Practical Rule	Practical Thresholds & Typical Values	Implication
Watchman FLX sizing (lobe/cap device -plug principle)	Size to landing-zone mean diameter with ~10–30% compression at release	If LZ = 22 mm, target device 24–27 mm (expect 10–30% compression)	Adequate compression provides stability & seal; excessive compression increases the risk of deformity
Amplatzer Amulet sizing (lobe/disc device -pacifier principle)	Lobe sized slightly larger than landing zone; disc must cover ostium with margin	Lobe typically +2–4 mm vs LZ; Disc ≥4–6 mm beyond ostium	In shallow or multilobed LAA, Amulet/LAmbre often advantageous due to disc sealing
Shallow LAA (depth <10–12 mm)	Favor disc-anchored devices; avoid devices needing deep coaxial seating	Depth 8–10 mm → prioritize disc coverage	Reduces protrusion/instability risk
Marked oval landing zone (ratio <0.8)	Increase oversizing within device limits; confirm seal on CT simulation	Consider +15–20% effective oversize	Improves circumferential apposition, lowers PDL risk
Complex/multilobed anatomy	Choose device with broad disc	Amulet/LAmbre	Enhances seal across irregular ostia
Risk to adjacent structures	Ensure disc distance 3–5 mm from MV/PV; reassess depth	Re-measure after simulated device plane	Prevents functional MR or PV flow issues
PDL on follow-up	Classify by maximal jet/contrast gap at ostium plane	Small <3 mm, Moderate 3–5 mm, Large >5 mm	>5 mm often clinically significant → consider OAC/redo; <5 mm may be acceptable (protocol-dependent)
DRT	Contrast CT with delayed phase; focal filling defect on device surface	Persistent low attenuation on delayed phase	Triggers antithrombotic escalation and close imaging follow-up
Thrombus exclusion	If arterial phase equivocal, rely on 60–90 s delayed	HU <~100 and no delayed fill = thrombus	Defer LAAO; treat and re-image

DRT, device-related thrombus; LZ, landing zone; OAC, oral anticoagulation; PDL, peri-device leak.

#### 3.4.1 Patient Selection and Pre-Procedural Planning

**Fig. 7.**
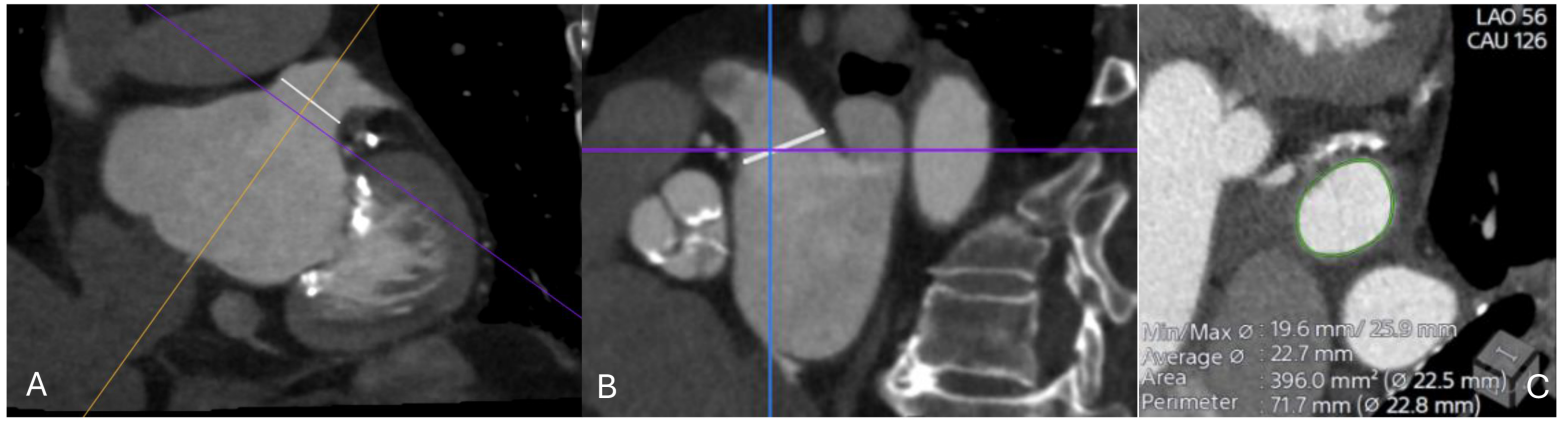
**CT imaging assessment of the left atrial appendage (LAA) prior to percutaneous closure**. (A) Oblique multiplanar reconstruction demonstrating the ostium of the LAA (white line), aligned along the true anatomical axis. (B) Orthogonal cross-sectional view showing the same ostial measurement (white line) in the perpendicular plane. (C) Cross-sectional reconstruction at the level of the ostium with automated contouring (green) and derived measurements of minimal and maximal diameters, average diameter, area, and perimeter (22.5–22.8 mm). These values are used for device sizing and procedural planning.

The first step in pre-procedural evaluation is exclusion of LAA thrombus. TOE remains widely used for this purpose, but contrast-enhanced CT with delayed imaging (typically 60–90 seconds post-contrast) has demonstrated high sensitivity and specificity for differentiating thrombus from slow flow. CT is particularly valuable for characterizing LAA morphology. It allows classification into common morphotypes (chicken wing, windsock, cactus, cauliflower), which have been linked to procedural feasibility and risk of residual leaks. CT characterizes LAA morphology and landing-zone geometry, as summarized in Tables 4,5 [111]. These measurements directly inform device sizing (Fig. 7). For example, the Watchman FLX typically requires oversizing of 10–20% relative to landing zone diameter, while the Amplatzer Amulet requires assessment of both ostial and landing zone diameters for appropriate sizing. Peripheral access evaluation is also essential, particularly in patients with peripheral vascular disease. CT allows assessment of iliofemoral access in the same dataset used for cardiac anatomy.

#### 3.4.2 CT vs. TOE in Guiding Device Selection

TOE offers real-time imaging and thrombus detection and traditionally has been used for the evaluation of the LAA and the proper device selection. However, TOE is limited by its two-dimensional nature and dependence on operator skill. CT, with its isotropic spatial resolution, enables precise reconstruction of the LAA ostium and landing zone in multiple planes, reducing under- or over-sizing, while can offer simulation of the device in MPR to confirm sealing [112]. It also delineates complex or multilobed appendages, where TEE may underestimate dimensions. In clinical practice, many centres use a hybrid approach with a TEE for thrombus exclusion and peri-procedural guidance, and CT for device planning. Increasingly, CT alone is being adopted for both pre-procedural planning and post-procedural follow-up, particularly as delayed phase protocols gain acceptance for thrombus evaluation.

#### 3.4.3 Follow-Up Imaging

Post-procedural imaging ensures adequate device endothelialisation and detection of peri-device leaks. TOE at 45 days remains the standard protocol in most centres, but CT offers complementary advantages as it can distinguish peri-device leak (contrast tracking into LAA) from peri-device residual space (non-opacified cavity without contrast). CT more reliably detects small leaks (<3 mm) than TOE however residual leaks detected by CT are lacking prognostic significance [113, 114]. Device-related thrombus, though rare, can be visualized with high confidence on CT using contrast and delayed acquisitions [115, 116] granting extension of the necessary antithrombotic therapy [117]. CT follow-up is also helpful for evaluating device deformation, protrusion into the left atrium, or impingement on nearby structures such as the mitral valve or pulmonary veins.

### 3.5 Atrial and Ventricular Septal Defect (ASD/VSD) Closures

#### 3.5.1 CT in Pre-Procedural Defect Sizing and Shunt Evaluation

While TOE remains the primary imaging modality for atrial and ventricular septal defects, cardiac CT provides complementary, high-resolution anatomical information in patients with suboptimal echo windows, complex anatomy, or prior interventions (Table 6) [118]. For ASDs, CT allows accurate measurement of the defect’s maximal diameter, shape (round vs. oval), and rims (superior vena cava, inferior vena cava, coronary sinus, and aortic rim). The presence of a deficient or absent rim—particularly at the aortic margin—affects feasibility of transcatheter closure. Typical device oversizing is by 20–40% relative to maximal defect diameter, depending on morphology [119]. CT also enables evaluation of associated anomalous pulmonary venous return, which can alter the management strategy [120].

**Table 6.** **CT measurements for ASD/VSD closure — what to measure, how, and clinical use**.

Measurement (CT)	How to measure (plane/method)	Practical thresholds/typical values	Planning & device implications
ASD maximal diameter (max/min/mean)	True interatrial septal plane via 3D MPR; align to fossa ovalis	Typical percutaneous range 6–38 mm; report max, min, mean	Size device to max diameter with ~20–40% oversizing or +2–6 mm (device/platform dependent)
ASD shape & ellipticity	Axial/orthogonal diameters at defect	Elliptical if short/long <0.8	Elliptical defects often need greater oversizing or devices with broader discs
Rim lengths (aortic, SVC, IVC, posterior)	Measure linear rim from defect edge to adjacent structures	Adequate ≥5 mm; aortic rim <5 mm = deficient	Deficient rims ↑ embolization/erosion risk; consider softer devices (e.g., Cardioform) or surgery if multiple rims are deficient
Septal tissue quality/thickness	Qualitative (thin vs robust) and thickness at rims	Thin/aneurysmal tissue (<~2–3 mm)	Favor larger disc coverage and cautious oversizing; avoid excessive radial force
Multifenestrated/aneurysmal IAS	3D overview; count fenestrations; measure aneurysmal sac	Multiple fenestrations or large aneurysm	Consider cribriform devices or single larger device spanning the aneurysmal segment
Pulmonary venous anatomy (ASD)	Survey PV drainage, especially right-sided	Rule out PAPVR	PAPVR may change management from percutaneous to surgical
VSD location	Classify: perimembranous, muscular, outlet, inlet	—	Determines approach (arterio-venous loop vs retrograde), sheath curve, and device type
VSD size (LV and RV sides)	Orthogonal diameters at LV and RV orifices; measure tunnel length if present	Percutaneous closure commonly ≤12–14 mm (center/device dependent)	Device waist typically +1–3 mm over maximal orifice; long/tunneled VSDs need elongated/duct-type devices
Distance to aortic valve (perimembranous/outlet VSD)	Shortest edge-to-cusp distance	Preferably ≥2–3 mm	<2–3 mm risks cusp impingement; consider smaller device or surgery
Distance to tricuspid subvalvular apparatus	Edge of defect to chordae/papillary structures	≥2–3 mm desirable	Close proximity risks TR or chordal injury; adjust device choice/approach
Shunt evaluation (contextual)	Ventricular volumetry; contrast timing	CT not primary for Qp:Qs	Use MRI for quantitative shunt; CT informs anatomy and access
Access & trajectory	3D path planning from femoral vein/artery; IAS thickness	Vein caliber ≥6–7 mm for large sheaths; IAS usually < 3–4 mm	Confirms feasibility and transseptal angle; optimizes sheath selection

IVC, inferior vena cava; LV, left ventricle; PAPVR, partial anomalous pulmonary venous return; SVC, superior vena cava; TR, tricuspid regurgitation.

For VSDs, CT can give important information regarding the location (peri-membranous, muscular, outlet, inlet), the size, and the relation to neighboring structures such as the aortic and tricuspid valves. Precise characterization of defect geometry is particularly valuable in muscular VSDs or in patients with prior surgical repair. 3D reconstructions help assess the trajectory for catheter-based closure. CT can also provide functional assessment of shunt severity when combined with ventricular volumetry or contrast timing analysis, though MRI remains the reference standard for Qp:Qs calculations [121].

#### 3.5.2 Role of CT in Guiding Device Selection and Post-Procedural Surveillance

Device choice depends on defect size, shape, and rim adequacy. CT provides reproducible measurements that minimize under- or over-sizing. The accepted indication of percutaneous ASD closure is a secundum defect with a maximal diameter below 38 mm and circumferential rim length over 5 mm [122]. For large, oval ASDs, devices with broader waist and discs (e.g., Amplatzer Septal Occluder or Figulla Flex II) may be more appropriate [123]. For muscular VSDs, CT aids in planning the delivery pathway, particularly in tortuous or aneurysmal defects [124].

Post-procedural CT can evaluate device position, residual shunts, and potential complications such as device embolization, erosion, or impingement on neighboring valves [125]. Multiplanar reformations allow detection of small peri-device leaks that may not be readily visualized on TEE, and 3D reconstructions help confirm device neo-endothelialization and integration and within the septum [126].

### 3.6 Other Structural Interventions

#### 3.6.1 Pulmonary Valve Interventions

Cardiac CT plays a central role in planning transcatheter pulmonary valve implantation (TPVI), particularly in patients with repaired congenital heart disease. CT accurately characterizes the right ventricular outflow tract (RVOT) morphology, conduit size, and degree of calcification [127]. Typical minimal diameters required for currently available devices (e.g., Melody, Sapien XT/3, Harmony) range from 16–29 mm, depending on device type [128]. CT also assesses the proximity of coronary arteries to the RVOT, as coronary compression during balloon inflation is a recognized complication [129]. Follow-up CT can detect stent fractures, conduit degeneration, and RVOT obstruction. Although MRI remains preferred for functional assessment of right ventricular volumes and regurgitation, CT is superior for anatomic evaluation in patients with metallic implants or contraindications to MRI [130].

#### 3.6.2 Cardiac CT for Paravalvular Leak (PVL) Closure

Paravalvular leaks represent a challenging complication after both surgical and transcatheter valve replacement. CT has become very important in defining leak morphology, which is often eccentric and irregular [131]. Multiplanar reformations along the prosthetic annulus allow precise localization, measurement of defect dimensions, and relationship to surrounding structures (Table 7). Pre-procedural CT planning enables selection of closure devices, appropriate sizing, and determination of access route (transseptal, retrograde aortic, or transapical) [132]. CT also helps exclude prosthetic instability or infection, which may contraindicate percutaneous repair. Post-procedural CT is useful for confirming device position and ruling out residual leaks, device embolization, or interference with prosthetic leaflet motion. In cases of multiple PVLs, CT can visualize the 3D distribution, guiding staged or combined closure strategies [133].

**Table 7.** **CT for paravalvular leak closure — measurement-driven planning**.

Measurement (CT)	How to measure (plane/method)	Practical thresholds/typical values	Planning & device implications
Leak location (clock-face mapping)	Reconstruct prosthetic annular plane; assign clock position (surgeon’s view)	—	Guides access route (transseptal vs retrograde vs transapical) and catheter orientation
Orifice size (max/min/mean)	Orthogonal diameters at annular orifice	Often 3–10+ mm; report shape	Round/oval → AVP II/IV; slit/crescentic → AVP III; plan ~30–50% oversizing vs max diameter
Leak arc length along annulus	Curvilinear measurement along annular plane	Short vs long (crescentic) arc	Long crescentic defects may require multiple plugs or elongated plugs for full seal
Tunnel/tract length & course	Centerline from annulus to atrial/ventricular exit; note bends	Long/angulated tracts	Favor duct-type/elongated plugs; avoid bulky discs that can impinge leaflets
Calcification & prosthesis–annulus interface	Qualitative and focal nodules	Heavy, asymmetric calcium	Choose conformable plugs; anticipate anchoring challenges; consider staged closure
Distance to prosthetic leaflets	Orifice to moving leaflet edge	Aim ≥2–3 mm clearance	Prevents leaflet impingement and prosthetic dysfunction
Aortic PVL: distance to coronary ostia	Orifice to ostial takeoff on 3D root	Prefer ≥5–10 mm	Close proximity → select low-profile plugs; confirm no ostial compromise
Mitral PVL: distance to LCx & LVOT	Orifice to LCx course and to LVOT	LCx clearance ≥5 mm preferred	Plan wire protection if close; avoid bulky devices near LVOT
Left atrial size & IAS for transseptal	LA dimensions; IAS thickness and puncture site	IAS usually <3–4 mm	Determines transseptal site and sheath curve for coaxial entry
Number of defects	3D survey around annulus	Single vs multiple	Multiple PVLs → staged or combined closure; tailor plug sizes per site
Post-closure surveillance	Contrast CT (arterial ± delayed) in annular plane	Residual jet gap: <3 mm small, 3–5 mm moderate, >5 mm large	>5 mm often clinically significant → consider additional plug/medical escalation

AVP, Amplatzer Vascular Plug; ADO, Amplatzer Duct Occluder; LCx, left circumflex artery; IAS, interatrial septum; OAC, oral anticoagulation.

## 4. Future Directions

The role of cardiac CT in structural heart interventions is expected to expand substantially over the next decade, driven by rapid technological innovation and integration with other imaging modalities. One key frontier is AI-based image analysis, which promises to automate anatomical segmentation, improve measurement reproducibility, and support real-time procedural planning. Early applications of machine learning have already demonstrated potential in automated annular sizing for TAVI [134] and in detection of peri-device leaks after LAAO [135]. While the promise is clear, widespread adoption remains limited by a lack of large, multicentre validation datasets, proprietary software variability, and regulatory challenges. Moreover, few AI systems have demonstrated consistent performance in patients with arrhythmias, heavy calcification, or motion artifacts—conditions frequently faced in structural heart populations. Future studies should therefore emphasize reproducibility across scanner vendors and image qualities, evaluate cost-effectiveness, and define clinically meaningful endpoints such as improved procedural planning efficiency or reduced complication rates.

Another emerging field is CT-derived functional imaging. Advances in dynamic and perfusion CT techniques allow estimation of flow patterns, myocardial perfusion, and even hemodynamic significance of shunts or leaks. This functional layer, when combined with detailed anatomical data, may provide a comprehensive one-stop assessment, reducing the need for multiple imaging tests [136]. Nevertheless, current evidence remains preliminary. Most perfusion CT studies involve small, single-centre cohorts [137] and use variable acquisition protocols that limit cross-study comparison. Dose exposure and contrast load also pose practical barriers to routine adoption. Comparative studies with cardiac MRI and echocardiography are needed to confirm the incremental diagnostic and prognostic value of CT-based functional assessment. Establishing standardized protocols and software platforms will be essential for consistent quantification of flow and perfusion parameters.

Equally important are efforts to reduce radiation and contrast exposure. Ultra-low-dose protocols [138], dual-energy CT [139], and photon-counting detectors are progressively lowering dose requirements while maintaining or even enhancing image quality [140]. Similarly, low-iodine contrast techniques could make CT safer for elderly patients with renal dysfunction, a population that represents the majority of candidates for structural interventions [141]. However, the clinical validation of these innovations remains incomplete. Ultra-low-dose acquisitions must demonstrate non-inferiority in anatomic accuracy for procedural planning [142], and dual-energy or photon-counting CT scanners remain costly and available primarily in tertiary centres [143]. Future prospective, multicentre trials comparing image quality, diagnostic precision, and clinical outcomes are warranted to support widespread implementation.

Finally, the integration of CT data with hybrid imaging platforms and procedural guidance systems is a promising perspective. Fusion of CT with fluoroscopy or echocardiography, as well as virtual or augmented reality applications, may enhance operator orientation and device navigation [144]. These innovations, combined with patient-specific simulation and 3D printing, have the potential to revolutionize preprocedural planning, training, and outcome prediction [145]. Despite these advances, integration into clinical workflows is currently limited by software compatibility, image registration accuracy, and the need for additional procedural hardware. Comparative studies are needed to quantify whether fusion imaging or virtual guidance translates into shorter procedure times, reduced contrast volume, or improved clinical outcomes. Collaboration between imaging specialists, engineers, and interventionalists will be critical to translate these tools from theory into daily practice.

In summary, the next phase of cardiac CT development will depend not only on technological refinement but also on rigorous clinical validation, cost-effectiveness, and standardization of acquisition and analysis protocols. The connection of AI-driven automation, functional assessment, dose optimization, and hybrid procedural integration is likely to redefine CT from a pre-procedural imaging tool into a comprehensive, real-time interventional companion.

## 5. Conclusion

CT has become a crucial imaging modality in the planning and follow-up of structural heart interventions. Its strengths lie in high-resolution 3D anatomy, reproducible measurements, and the ability to guide critical decisions such as device sizing, access route selection, and risk prediction. Despite limitations related to radiation, contrast use, and susceptibility to motion artifacts, continuous technical refinements and growing operator expertise have significantly minimise these challenges. CT is no longer a supplementary tool but rather a cornerstone of structural heart disease management. As the landscape of structural interventions expands, CT will remain at the forefront, ensuring procedures are not only feasible but also optimized for safety and long-term success.
